# PLACE OF DEATH AND CARE SATISFACTION AMONG THE HEALTH AND RETIREMENT STUDY DECEDENTS

**DOI:** 10.1093/geroni/igac059.1401

**Published:** 2022-12-20

**Authors:** Ayse Malatyali, Zahra Rahemi, Tom Cidav, Cheryl Dye, Olga Jarrín, Christopher McMahan

**Affiliations:** University of Central Florida, Orlando, Florida, United States; Clemson University, Greenville, South Carolina, United States; Johns Hopkins University, Baltimore, Maryland, United States; Clemson University, Clemson, South Carolina, United States; Rutgers, The State University of New Jersey, New Brunswick, New Jersey, United States; Clemson University, Clemson, South Carolina, United States

## Abstract

The place of care at the time of death can influence the satisfaction, type, and cost of end-of-life care. This study investigated factors associated with place of death among older adults in the Health and Retirement Study (Exit files 2002-2018) with cognitive impairment (n=3,102). Black and Hispanic participants were more likely to die in the hospital (OR=1.80, 1.47) and less likely to die in a nursing home (OR=0.54, 0.37) than white and non-Hispanic participants. Hispanic participants were also 50% more likely to die at home than non-Hispanics. Compared to other places, participants who died at home were 43% more satisfied, and participants who died at nursing homes were 32% less satisfied with the care they received. There was no significant effect of ethnicity on the relationship between place of death and satisfaction with care. Investigating the moderating role of other demographic factors can shed more light on this relationship.

